# Transcriptional regulation of *cpsQ*‐*mfpABC* and *mfpABC* by CalR in *Vibrio parahaemolyticus*

**DOI:** 10.1002/mbo3.470

**Published:** 2017-03-20

**Authors:** He Gao, Lingyu Zhang, George Osei‐Adjei, Wenhui Yang, Dongsheng Zhou, Xinxiang Huang, Huiying Yang, Zhe Yin, Yiquan Zhang

**Affiliations:** ^1^ State Key Laboratory for Infectious Disease Prevention and Control National Institute for Communicable Disease Control and Prevention Chinese Centre for Disease Control and Prevention Beijing China; ^2^ School of Medicine Jiangsu University Zhenjiang Jiangsu China; ^3^ State Key Laboratory of Pathogen and Biosecurity Beijing Institute of Microbiology and Epidemiology Beijing China

**Keywords:** CalR, *cpsQ‐mfpABC*, regulation, *Vibrio parahaemolyticus*

## Abstract

The *cpsQ‐mfpABC* locus is transcribed as two operons, i.e., *cpsQ‐mfpABC* and *mfpABC*, in *Vibrio parahaemolyticus*, and both of them are all required for biofilm formation. CalR belongs to the LysR‐type transcriptional regulator family, and was originally identified as a repressor of the swarming motility and T3SS1 genes expression in *V*. *parahaemolyticus*. In the present work, a combination of qRT‐PCR, primer extension, LacZ fusion expression, electrophoretic mobility shift assay, and DNase I footprinting assays were employed to elucidate the regulatory mechanisms of *cpsQ‐mfpABC* and *mfpABC* by CalR. One transcription start site for each operon was detected and their activities were activated by CalR. His‐CalR protected two DNA regions upstream of *mfpABC* against DNase I digestion, but no binding sites were detected in the promoter region of *cpsQ‐mfpABC*, suggesting a direct and an indirect regulatory manner for *mfpABC* and *cpsQ‐mfpABC* transcription by CalR, respectively. Collectively, the results presented here confirmed a new physiological role for CalR that acts as an activator for *cpsQ‐mfpABC* and *mfpABC* transcription.

## 1 Introduction

*Vibrio parahaemolyticus* is a Gram‐negative, halophilic bacterium that naturally inhabits estuarine, marine, and coastal environments. The overwhelming majority of *V*. *parahaemolyticus* strains are environmental and nonpathogenic, only a small proportion of isolates are pathogenic to humans. Virulent *V*. *parahaemolyticus* strains are capable of causing three different types of diseases that are acute gastroenteritis, wound infections, and septicemia (Broberg, Calder, & Orth, 2011). The acute gastroenteritis is the most common type and is mostly caused by consumption of raw or undercooked seafood. In the last years, *V*. *parahaemolyticus* was recognized as a leading cause of seafood‐associated bacterial gastroenteritis (Su & Liu, 2007).

Virulent *V*. *parahaemolyticus* strains expresses a number of virulence factors involved in pathogenicity including thermostable direct hemolysin (TDH), TDH‐related hemolysin (TRH), two type III secretion systems (T3SS1 and T3SS2), two type VI secretion systems (T6SS1 and T6SS2), as well as some adhesins (Makino et al., 2003). Current findings show that expression of the virulence factors is tightly regulated by numerous regulators or environmental growth conditions. For instance, transcription of T3SS1‐related genes is induced by ExsA (Zhou, Konkel, & Call, 2010), calcium and iron (Gode‐Potratz, Chodur, & Mccarter, 2010), whereas it is repressed by ToxR (Whitaker, Parent, Boyd, Richards, & Boyd, 2012), H‐NS (Sun et al., 2014; Zhang et al., 2016), CalR (Gode‐Potratz et al., 2010), as well as the small RNA Spot 42 posttranscriptionally (Tanabe, Miyamoto, Tsujibo, Yamamoto, & Funahashi, 2015). *V*. *parahaemolyticus* quorum sensing (QS) system also appears to have regulatory effect on T3SS1 expression (Henke & Bassler, 2004). Consequently, a variety of mechanisms are employed to control production of the virulence factors in *V*. *parahaemolyticus*.

The *cpsQ‐mfpABC* (VPA1445‐1448) locus contains two operons: *cpsQ‐mfpABC* and *mfpABC* (Zhou et al., 2013). The *cpsQ* encodes a c‐di‐GMP‐binding regulatory protein that directly and positively regulates the expression of *cps* loci encoding capsular polysaccharide, which is a major component of biofilm matrix of *V*. *parahaemolyticus* (Ferreira, Chodur, Antunes, Trimble, & Mccarter, 2012). The *mfpABC* operon encodes the membrane fusion proteins that are contributors of biofilm formation in *V*. *parahaemolyticus*, and *mfp* mutants showed a severe defect in biofilm formation (Enos‐Berlage, Guvener, Keenan, & Mccarter, 2005). We reported previously that transcription of *cpsQ‐mfpABC* and *mfpABC* are regulated by AphA and OpaR, the two sole master regulators of QS in *V*. *parahaemolyticus* (Zhou et al., 2013). Herein, we investigated the transcriptional regulation of the two operons by CalR in *V*. *parahaemolyticus*.

CalR (VP0350) belongs to the LysR‐type transcriptional regulator family, and is annotated as an LeuO homologue. Besides inhibition of the expression of T3SS1, Gode‐Potratz et al., previously demonstrated that CalR also has the ability to repress *V*. *parahaemolyticus* swarming motility (Gode‐Potratz et al., 2010). This study reported that CalR can activate *cpsQ‐mfpABC* and *mfpABC* transcription in an indirect and a direct manner, respectively.

## 2 Materials and Methods

### 2.1 Bacterial strains

The *V*. *parahaemolyticus* RIMD 2210633 was used as the wild‐type (WT) strain (Makino et al., 2003). The *calR* deletion mutant (*ΔcalR*) was generated as previously described (Sun et al., 2012; Zhang et al., 2012). Briefly, the 405 and 426 bp DNA regions upstream and downstream of *calR* were amplified by PCR, purified, and used as templates to create an 801 bp deletion construct that was subsequently inserted between the *Pst* I and *Sph* I sites of pDS132. After being verified by DNA sequencing, the recombinant vector was transformed into *Escherichia coli* S17‐1 (pir), and then transferred into WT by conjugation. The mutant strain was selected, using resistance to 10% sucrose and sensitivity to 5 μg/ml chloramphenicol, and further verified by PCR.

For complementation of the *ΔcalR* (Sun et al., 2012), a PCR‐generated DNA fragment containing the *calR* coding region together with an upstream synthetic ribosome‐binding site (RBS) was cloned into the pBAD33 vector harboring an arabinose P_BAD_ promoter and a chloramphenicol resistance gene. The recombinant plasmid pBAD33‐*calR* was verified by DNA sequencing, and subsequently transformed into *ΔcalR*, yielding the complemented mutant strain *ΔcalR/*pBAD33‐*calR*. For controls, the empty vector pBAD33 was also transformed into WT and *ΔcalR* to generate WT/pBAD33 and *ΔcalR/*pBAD33. All the primers used in the present work were listed in Table 1.

**Table 1:** Oligonucleotide primers used in this study

Type of analysis and primer	Sequences (5′‐3′)
Construction of mutants	
*calR*‐A	GTAGCTGCAGGCAGATTATTTGACTGATACGC
*calR*‐B	GTTCGCAAATGGGAAGTCTCTCATCGCATCTTTCTTCTC
*calR*‐C	GAGAAGAAAGATGCGATGAGAGACTTCCCATTTGCGAAC
*calR*‐D	GTGAGCATGCTACTTACCTTTTGGCTTACAG
Construction of complementary strain	
*calR*‐HP‐F/R	GCGGTCGACAGGAGGAATTCACCATGTTAGAGAAGAAAGATG/GCGAAGCTTTTATTTTGATGCGACCAC
Protein expression	
*calR*‐P‐F/R	GCGGGATCCATGTTAGAGAAGAAAGATG/GCGAAGCTTTTATTTTGATGCGACCAC
qRT‐PCR	
*cpsQ*‐RT‐F/R	GCCTGAAATCCTAATGCTC/AGTGTCAGAAGGTGTATCAAC
*mfpA*‐RT‐F/R	GCGGGCAATGATCGTCTAAC/TCACCTGAACCTGCGACAAG
Primer extension	
*cpsQ*‐PE‐R	/GATTTCAGGCTTTTCCGTGTAC
*mfpA*‐PE‐R	/ATTCCCTCTGGCTTATTTATTG
LacZ fusion	
*cpsQ‐lacZ*‐F/R	GCGCGTCGACCAGACGGGCATTGATAAG/GCGCGAATTCCATTAGGATTTCAGGCTTTT
*mfpA‐lacZ*‐F/R	GCGCGTCGACTTATGACTTAGATACCGAA/GCGCGAATTCCGAAATCAGCGATATTGTTG
EMSA	
*cpsQ‐*EMSA‐F/R	GTTCCAGCAATACTGACTAAGC/GATTTCAGGCTTTTCCGTGTAC
*mfpA‐*EMSA‐F/R	TAGGACGCAAGCCACAAG/CGAAATCAGCGATATTGTTG
16S rDNA‐RT‐F/R	GACACGGTCCAGACTCCTAC/GGTGCTTCTTCTGTCGCTAAC
DNase I footprinting	
*cpsQ*‐FP‐F (M13F)	GTAAAACGACGGCCAGTCCTAACTAATTTAGTGCA
*cpsQ*‐FP‐R (M13R)	CAGGAAACAGCTATGAC TTCAGGCTTTTCCGTGTAC
*mfpA*‐FP‐F (M13F)	GTAAAACGACGGCCAGTTAGGACGCAAGCCACAAG
*mfpA‐*FP‐R (M13R)	CAGGAAACAGCTATGAC CGAAATCAGCGATATTGTTG
M13F‐FAM	GTAAAACGACGGCCAGT
M13R‐HEX	CAGGAAACAGCTATGAC

### 2.2 Growth conditions

For *V*. *parahaemolyticus* cultivation, bacteria were grown in complete HI broth (2.5% Bacto heart infusion [BD Bioscience]) at 37°C with shaking at 250 rpm. We designed three‐step cultivation of bacterial cells for the following gene regulation assays: firstly, the glyceric stock of bacteria was inoculated into 5 ml of HI broth and allowed to grow overnight; secondly, the overnight cell cultures were diluted 1:50 into 15 ml of fresh HI broth, and grown to reach an OD_600_ value of about 1.0–1.2; thirdly, the bacterial cell cultures in the second step were diluted 1:1,000 into 15 ml of HI broth for the third‐round growth, and were harvested at an OD_600_ value of about 1.0–1.2. When required, the culture medium was supplemented with 50 μg/ml gentamicin, 5 μg/ml chloromycetin, or 0.1% arabinose.

### 2.3 RNA isolation and quantitative real‐time PCR (qRT‐PCR)

Total RNAs were extracted, using the TRIzol reagent (Invitrogen). RNA quality and quantity were monitored by agarose gel electrophoresis and spectrophotometry, respectively (Sun et al., 2012; Zhang et al., 2012). The contaminant genome DNA in the total RNAs was removed by using the Ambion's DNA‐free^™^ Kit. cDNAs were generated, using 3−8 μg of RNA and 3 μg of random hexamer primers. The SYBR Green qRT‐PCR assay was performed and analyzed as previously described (Gao et al., 2011). The experiment was performed with at least three independent cultures and RNA preparations.

### 2.4 Primer extension assay

For the primer extension assay (Sun et al., 2012; Zhang et al., 2012), 3−10 μg of total RNAs was annealed with 1 pmol of 5′‐ ^32^P‐labeled reverse oligonucleotide primer to generate cDNAs, using a Primer Extension System (Promega) according to the manufacturer's instructions. The same labeled primer was used for sequencing with the AccuPower & Top DNA Sequencing Kit (Bioneer). The primer extension products and sequencing materials were concentrated and analyzed in an 8 mol/L urea‐6% polyacrylamide gel electrophoresis, and the results were detected by autoradiography with the Fuji Medical X‐ray film.

### 2.5 LacZ fusion and β‐galactosidase assay

The promoter DNA region of each indicated gene was amplified and cloned into the corresponding restriction endonuclease sites of low‐copy‐number plasmid pHRP309 that harbors a gentamicin resistance gene and a promoterless *lacZ* reporter gene (Parales & Harwood, 1993). After being verified by DNA sequencing, the recombinant pHRP309 plasmid was transferred into *V*. *parahaemolyticus* strains. An empty pHRP309 plasmid was also introduced into each strain tested as the negative control. The *V*. *parahaemolyticus* strains transformed with recombinant or empty pHRP309 plasmids were grown as above to measure the β‐galactosidase activity in cellular extracts using the β‐Galactosidase Enzyme Assay System (Promega) according to the manufacturer's instructions.

### 2.6 Preparation of 6× His‐tagged CalR (His‐CalR) protein

The entire coding region of *calR* was amplified, purified, and cloned between *BamH*I and *Hind*III sites of plasmid pET28a (Novagen). The recombinant plasmid encoding His‐CalR was then transformed into *E. coli* BL21λDE3 cells. Expression of His‐CalR was induced by adding 1 mmol/L IPTG (isopropyl‐b‐D‐thiogalactoside) and purified under native conditions with nickel‐loaded HiTrap Chelating Sepharose columns (Amersham) (Sun et al., 2012). Briefly, cells were collected by centrifugation, and then resuspended in 10 ml of 57 mmol/L sodium phosphate buffer, 500 mmol/L NaCl, 5 mmol/L imidazole, pH 8.0. After being disrupted by a cell cracker at >1000 psi, the insoluble material was pelleted by centrifugation at 10,000 rpm for 30 min under 4°C. The clarified supernatant was loaded onto a 5 ml nickel−chelating column, then washed with five column volumes of wash buffer, and eluted with an imidazole gradient. The eluant containing His‐CalR protein was dialyzed against 0.02 mol/L PBS (41 mmol/L Na_2_HPO_4_, 5 mmol/L NaH_2_PO_4_, 145 mmol/L NaCl, and 20% glycerol, pH 8.0), and concentrated to a final concentration of about 0.3−0.6 mg/ml. The purified protein was stored at −80°C, and the protein purity was confirmed by SDS‐PAGE.

### 2.7 Electrophoretic mobility shift assay (EMSA)

The promoter DNA region of each target gene was amplified by PCR. For EMSA (Sun et al., 2012; Zhang et al., 2012), DNA binding was performed in a 10 μl reaction volume containing binding buffer (1 mmol/L MgCl_2_, 0.5 mmol/L EDTA, 0.5 mmol/L DTT, 50 mmol/L NaCl, 10 mmol/L Tris‐HCl [pH 7.5] and 10 mg/ml salmon sperm DNA), 100–200 ng target promoter DNA, and increasing amounts of His‐CalR. After incubation at room temperature for 20 min, the products were loaded onto a native 6% (w/v) polyacrylamide gel, and electrophoresed in 0.5× TBE buffer for about 90 min at 200 V. After staining with ethidium bromide (EB) dye, the gel was examined with a UV transilluminator.

### 2.8 DNase I footprinting assay

DNase I footprinting assays were carried out similar to the method described by Zianni, Tessanne, Merighi, Laguna, and Tabita (2006). Briefly, a DNA fragment of promoter DNA region of each indicated genes was PCR amplified, using the primers target‐FP‐F (M13F) and target‐FP‐R (M13R) with *ExTaq* DNA polymerase. After being purified, the amplicon was used as the template for labeling the probes with different primer pairs: M13F‐FAM and target gene‐FP‐R (M13R) for preparation of 6‐carboxyfluorescein (FAM)‐labeled coding strand, and target gene‐FP‐F (M13F) and M13R‐HXE for preparation of 5′‐Hexachloro‐fluorescein phosphoramidite (HEX)‐labeled noncoding strand. The PCR products were purified, using the Qiaquick columns (Qiagen) and quantified with a NanoDrop 2000 (Thermo). Approximately, 350 ng of the purified FAM/HEX‐labeled DNA fragments were incubated with increasing amounts of His‐CalR in a final 10 μl reaction volume containing the binding buffer used in EMSA. After incubation for 30 min at room temperature, 10 μl of Ca^2+^/Mg^2+^ solution (5 mmol/L CaCl_2_ and 10 mmol/L MgCl_2_) was added, followed by further incubation for 1 min at room temperature. The optimized RQ1 RNase‐Free DNase I (Promega) was then added to the reaction mixture, and the mixture was incubated at room temperature for 30–50 s. The reaction was stopped by adding 9 μl of stop solution (200 mmol/L NaCl, 30 mmol/L EDTA, and 1% SDS). The digested DNA samples were extracted with a Beaver Beads^™^PCR Purification Kit (Beaver) according to the manufacturer's instructions, and the sample pellets were dissolved in 15 μl modified water (HiDi: water: 600 LIZ=90:60:1). For sequencing, the BigDye^®^ Terminator v3.1 Cycle Sequecing Kits (ABI) was used. The volume of each sequencing reaction was enlarged to 20 μl that contains 10 ng of target promoter region as template, 3.2 pmol of sense or antisense primer as the sequencing primer, and 8 μl of BigDye reaction mix (BigDye: 5× buffer = 1:3). After predenaturation at 96°C for 1 min, PCR amplification was conducted at 25 cycles of denaturation at 96°C for 10 s, annealing at 50°C for 5 s and extension at 60°C for 4 min. The sequencing samples were precipitated with the same method as above, and then dissolved in 10 μl HiDi and 1 μl 600 LIZ. The digested DNA fragments were analyzed, using ABI 3500XL DNA Genetic analyzer with GeneMarker software 2.2. The sequencing products were surveyed with Sequence Scanner software v1.0.

### 2.9 Experimental replicates and statistical methods

For the LacZ fusion and qRT‐PCR assays, experiments were performed with at least three independent bacterial cultures, and values were expressed as mean ± standard deviation (SD). Paired Student's *t* test was used to calculate statistically significant differences, *p* <0 .01 was considered to indicate statistical significance. The presented data of primer extension and DNase I footprinting assays were done with at least two independent biological replicates.

## 3 Results

### 3.1 CalR activates *cpsQ‐mfpABC* and *mfpABC* transcription

**Figure 1:**
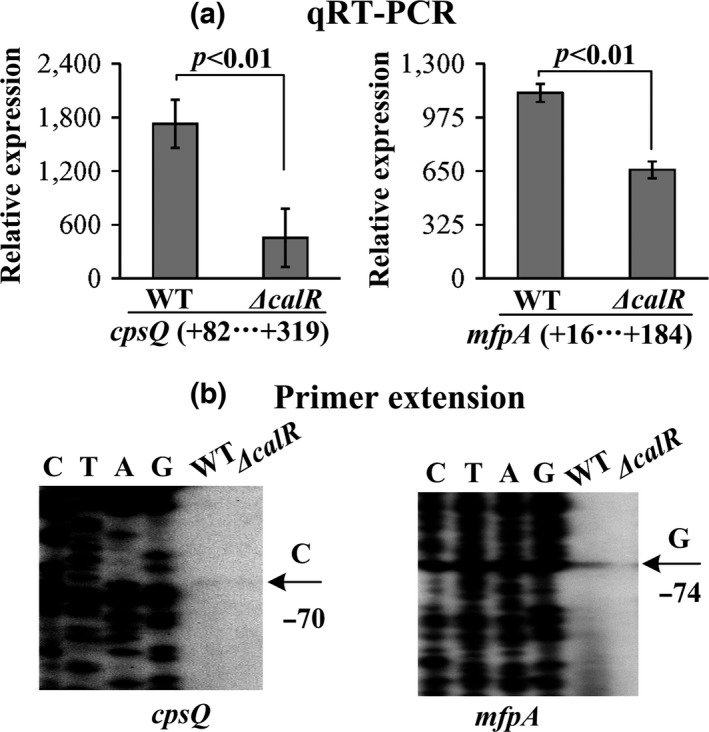
Transcription of *cpsQ‐mfpABC* and *mfpABC* were positively regulated by CalR. The minus and positive numbers indicated the nucleotide positions upstream and downstream of indicated genes. (a) qRT‐PCR. The relative mRNA levels of each target gene were compared between *ΔcalR* and WT. (b) Primer extension. An oligonucleotide primer was designed to be complementary to the RNA transcript of each target gene. The primer extension products were analyzed with an 8 mol/L urea‐6% acrylamide sequencing gel. The transcriptional start sites were indicated by arrows with nucleotide and position. Lanes C, T, A, and G represent the Sanger sequencing reactions

The qRT‐PCR assay (Figure 1a) was conducted to detect the mRNA levels of the target genes in WT and *ΔcalR*. The results indicated that the mRNA levels of both *cpsQ* and *mfpA* was evidently decreased in *ΔcalR* relative to WT, suggesting a positive regulation manner of *cpsQ‐mfpABC* and *mfpABC* by CalR in *V*. *parahaemolyticus*. In order to further disclose the positive regulation of *cpsQ‐mfpABC* and *mfpABC* by CalR, the primer extension assay (Figure 1b) was carried out. This assay detected only one transcription start site for each of the two genes, and their activities were under the positive control of the CalR protein. Collectively, transcription of *cpsQ‐mfpABC* and *mfpABC* is activated by CalR in *V*. *parahaemolyticus*.

### 3.2 CalR activates the promoter activities of *cpsQ‐mfpABC* and *mfpABC*

**Figure 2:**
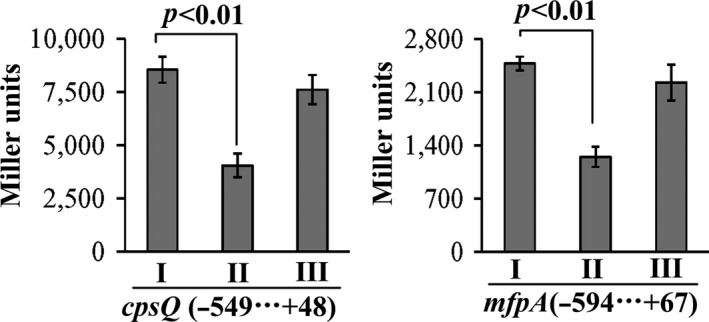
CalR activates the promoter activities of *cpsQ‐mfpABC* and *mfpABC*. The target promoter DNA region of each target genes was cloned into the pHRP309 vector containing a promoterless *lacZ* gene. Thereafter, the recombinant plasmids were transformed into WT/pBDA33, *ΔcalR*/pBDA33, and *ΔcalR/* pBDA33‐*calR*, respectively, to determine the β‐galactosidase activity (miller units) in the cellular extracts. The minus and positive numbers represent the nucleotide positions upstream and downstream of indicated gene. I, II, and III represent WT/pBDA33, *ΔcalR*/pBDA33 and *ΔcalR/* pBDA33‐*calR*, respectively

The recombinant *lacZ* vector that contains the indicated target promoter region and promoterless *lacZ* gene was transformed into WT/pBDA33, *ΔcalR*/pBDA33 and *ΔcalR*/pBDA33‐*calR*, respectively, to test the action of CalR on the promoter activity of each gene in these three strains. As shown in Figure 2, the β‐galactosidase activity (Miller units) of each of the two target genes was significantly decreased in *ΔcalR*/pBDA33 relative to that in WT/pBDA33 or *ΔcalR/*pBDA33‐*calR*, but it was manifested almost at the same levels in WT/pBDA33 and *ΔcalR/*pBDA33‐*calR*. These results confirmed that transcription of *cpsQ‐mfpABC* and *mfpA* is under positive control of CalR.

### 3.3 Analysis of DNA‐binding activity of His‐CalR to the target promoters

**Figure 3:**
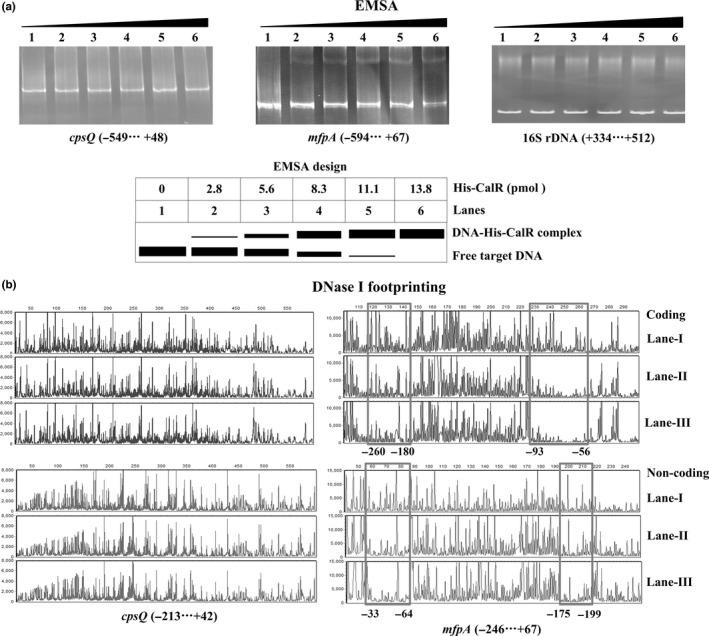
DNA‐binding activity of His‐CalR to the target promoters. The negative and positive numbers indicated the nucleotide positions relative to the translation start site (+1) of target genes, respectively. (a) EMSA. The promoter DNA regions of target genes were incubated with increasing amounts of purified His‐CalR protein, and then subjected to 6% (w/v) polyacrylamide gel electrophoresis. The DNA bands were visualized by EB staining. Shown below the EMSA results is the EMSA design. (b) DNase I footprinting. The promoter DNA fragments of *cpsQ‐mfpABC* and *mfpABC* were labelled with FAM, incubated with increasing amounts of purified His‐CalR (Lanes‐I, II, and III containing 0, 5.52, and 11.04 pmol, respectively), and then subjected to DNase I footprinting assay. The fragments length was analyzed, using an ABI 3500XL DNA analyzer. The footprint regions were boxed and marked with positions

The entire promoter DNA regions of *cpsQ‐mfpABC* and *mfpABC* were amplified, purified, and subjected to EMSA with purified His‐CalR protein (Figure 3a). The results showed that His‐CalR was unable to bind to the upstream DNA fragment of *cpsQ‐mfpABC*, but could bind to *mfpABC* promoter in a dose dependent manner. By contrast, His‐CalR could not bind to the 16S rDNA at all tested amounts as the negative control. As further determined by DNase I footprinting (Figure 3b), His‐CalR protected two different DNA regions upstream of *mfpA* against DNase I digestion that were considered as the CalR sites; however, no CalR sites were detected in the promoter region of *cpsQ‐mfpABC*. Taken together, CalR activates *mfpABC* transcription in a direct manner, but it appears to positively regulate *cpsQ‐ mfpABC* transcription in an indirect manner.

### 3.4 Promoter structure of *mfpABC*

**Figure 4:**
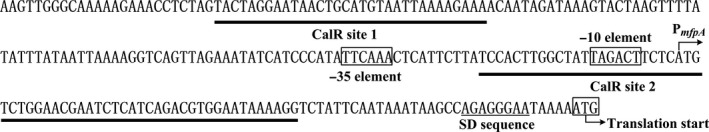
Organization of *mfpABC* promoter‐proximal DNA region. The DNA sequence was derived from RIMD 221063. The bent arrows indicate the transcriptional and translational start sites, respectively. The −10/−35 elements are enclosed in boxes. The SD box and CalR sites are underlined with solid lines

Collection of the data presented here enabled us to reconstruct the organization of CalR‐dependent *mfpABC* promoter that contains the translation/transcription start sites, promoter −10 and −35 elements, CalR sites, and Shine‐Dalgarno (SD) sequences (ribosomal‐binding sites) (Figure 4).

## 4 Discussion

The *mfp* loci express the CpsQ regulator and the MfpABC transporter, and both of them are required for biofilm formation (Enos‐Berlage et al., 2005; Ferreira et al., 2012). However, the detailed roles of CpsQ and MfpABC in other cellular pathways and their expression regulatory mechanisms are still obscure. We previously demonstrated that transcription of *cpsQ‐mfpABC* and *mfpABC* are repressed and activated by AphA and OpaR, respectively, and thereby the transcriptional levels of *cpsQ‐mfpABC* and *mfpABC* enhance gradually with transition from low cell density to high cell density, suggesting CpsQ and MfpABC may play roles at middle/late stages of growth and pathogenesis (Zhou et al., 2013). In this study, a set of experiments were used to investigate the regulation of *cpsQ‐mfpABC* and *mfpABC* by CalR in *V*. *parahaemolyticus*. We found that transcription of *cpsQ‐mfpABC* and *mfpABC* was activated by CalR in an indirect and a direct manner, respectively. Therefore, we reconstructed the structural organization of *mfpABC* promoter‐proximal DNA region by collecting of core promoter −10 and −35 elements, SD sequence, translation/transcription start sites, and CalR‐binding sites. As shown in Figure 4, the CalR site 1 was upstream of the promoter −35 element, while the CalR site 2 overlaps the core promoter‐10 and transcription start of *mfpABC*. This is an abnormal transcriptional stimulation mode (Ishihama, 2000). However, the binding site of AphA, which acts as a transcriptional repressor of *mfpABC*, is located from −129 to −84 upstream of *mfpABC* (Zhou et al., 2013). Additionally, CalR site 2 is located from −93 to −33 upstream of *mfpABC*. There are 10 bp overlaps between AphA site and CalR site 2. Therefore, we surmise that CalR can block the binding activity of AphA to the promoter region, thereby activating the *mfpABC* transcription. CalR is annotated as an LeuO homologue. Studies in *Salmonella* demonstrated that LeuO functions as an H‐NS antagonist through dispalcing H‐NS binding to target promoters (De La Cruz et al., 2007; Dillon et al., 2012). H‐NS is a histone‐like nucleoid structure protein, and has been described as a transcriptional repressor (Bouffartigues, Buckle, Badaut, Travers, & Rimsky, 2007). H‐NS shows preference for binding to A/T‐rich and curved DNA sequences (Bouffartigues et al., 2007), and the 234 bp upstream DNA fragment of *mfpABC* is a high A + T content (>69%). Therefore, we hypothesize that *mfpABC* would be under direct and negative control of H‐NS, and CalR may antagonize H‐NS‐dependent repression of *mfpABC*. We will characterize these two hypothesises in our future studies.

## Conflict of Interest

None declared.
